# Supplementary material for: The adaptive large language models for vaccine prediction: A novel approach to vaccine demand prediction with engineered deviation prompts
Source: PLOS Digit Health. 2026 Mar 9;5(3):e0001273. doi: 10.1371/journal.pdig.0001273 (PMC12970898; doi:10.1371/journal.pdig.0001273)
Supplement: S1 Appendix — (DOCX) [file pdig.0001273.s001.docx]

**Appendix A: Prompt Engineering Framework for Vaccine Demand Prediction**

This section provides the full pseudo-code for the ALLMVP model.

================================================================

# PSEUDOCODE: ADAPTIVE LARGE LANGUAGE MODEL FOR VACCINE PREDICTION (ALLMVP)

# Step 1: Data Acquisition and Preprocessing

Load raw vaccine distribution data from multiple clinics. Preprocess data to generate clean structured format:

Vaccine_Flow_Data = {

vaccine_1: {'date_day': dose_count, ...} # date_day format: YYYY-MM-DD. Please note that dates may be repeated because the data comes from different clinics, so the same vaccine may be administered at multiple locations on the same day.

vaccine_2: {'date_day': dose_count, ...} # dose_count represents the amount of vaccine used that day, for example, 5

vaccine_3: {'date_day': dose_count, ...}

...

}

# Step 2: Process Each Vaccine Type

FOR each vaccine_name, daily_data in Vaccine_Flow_Data: # eg. vaccine_name = A1, vaccine_value = Vaccine_Flow_Data['A1'] = {'date_day': vaccine_num, ...}

For each month in range 2018 to 2022:

# 2.1 Generate Daily Summary (Consolidate Multi-Clinic Data)

vaccine_daily_summary = CALL GPT-4_API(

prompt = daily summary prompt,

input_data = vaccine_value

)

# Output: {"2014-01-01": total_doses, ...} # Unique dates

# 2.2 Generate Monthly Aggregation

vaccine_monthly_summary = CALL GPT-4_API(

prompt = monthly summary promt,

input_data = vaccine_value

)

# Output: {"2014-01": monthly_total, ...}

# 2.3 Model Forecasting

predicted_value = CALL GPT-4_API(

prompt = times series prediction prompt,

input_data = vaccine_daily_summary, vaccine_monthly_summary

)

# Output: {"2018-01": predicted_doses}

# 2.4 Demand Correction (Calibration Step)

correction_value = CALL GPT-4_API(

prompt = real demand prompt,

input_data = predicted_value, true_value, history_value

)

# Output: {"2018-01": correction_ratio}

# 2.5 Generate Final Output

final_data_value = CALL GPT-4_API(

prompt = vaccine prompt,

input_data = predicted value, correction value

)

# Output: {"month": "2018-01", "predicted_value": predicted_value, "final_predicted_value": final_predicted_value, "correction_value": correction_value}

# Step 3: Output Results

PRINT formatted_output FOR vaccine_name:

Month, Predicted_Value, Final_Predicted_Value, Correction_Value

2018-01, 1525, 1525, 0.8

2018-02, 1676, 1341, 0.49

...

================================================================

The implementation deploys model="gpt-4.1" with controlled generation parameters (temperature=0.2, max_tokens=1000, frequency_penalty=0.0), system prompts is "You are a helpful assistant for text processing", the formal definition of user prompts is as follows:

(1) Daily Summary Prompt Template

As a vaccine usage data analyst, your task is to process raw daily vaccine usage records. Clean and merge the data, combining records for the same vaccine on the same day into a single total. Finally, organize the data into a complete table and save it to a CSV file.

Raw daily vaccine usage records:

<Vaccine Usage Raw Records>

A1,1,2014-01-02

A1,1,2014-01-03

A1,1,2014-01-07

....

</Vaccine Usage Raw Records>

Process the data according to the following steps:

Carefully inspect the raw records to remove any empty rows, duplicate entries, or obviously erroneous data.

Iterate through all records. For entries corresponding to the same vaccine on the same day, sum and combine the usage quantities.

Organize the merged data into a complete table with three columns: Vaccine Name, Total Usage Quantity, and Date.

After processing, save the table data to a CSV file. Use commas as delimiters. The first line should be the header (Vaccine Name,Total Usage Quantity,Date). Subsequent lines should contain each merged data entry.

Write the processed table data within the <Processed Table> tags. Use plain text format with comma-separated columns and newline-separated rows. The first row must be the header.

<Processed Table>

Vaccine Name,Total Usage Quantity,Date

A1,10,2014-01-02

A1,12,2014-01-03

A1,13,2014-01-07

....

</Processed Table>

(2) Monthly Summary Prompt Template

As a vaccine usage data analyst, your task is to process raw daily vaccine usage records. Clean and merge the data, combining records for the same vaccine on the same month into a single total. Finally, organize the data into a complete table and save it to a CSV file.

Raw daily vaccine usage records:

<Vaccine Usage Raw Records>

A1,1,2014-01-02

A1,1,2014-01-03

A1,1,2014-01-07

....

</Vaccine Usage Raw Records>

Process the data according to the following steps:

Carefully inspect the raw records to remove any empty rows, duplicate entries, or obviously erroneous data.

Iterate through all records. For entries corresponding to the same vaccine on the same month, sum and combine the usage quantities.

Organize the merged data into a complete table with three columns: Vaccine Name, Total Usage Quantity, and Date.

After processing, save the table data to a CSV file. Use commas as delimiters. The first line should be the header (Vaccine Name,Total Usage Quantity,Date). Subsequent lines should contain each merged data entry.

Write the processed table data within the <Processed Table> tags. Use plain text format with comma-separated columns and newline-separated rows. The first row must be the header.

<Processed Table>

Vaccine Name,Total Usage Quantity,Date

A1,20,2014-02

A1,32,2014-03

A1,33,2014-04

....

</Processed Table>

(3) Time Series Prediction Prompt Template

You are a vaccine usage data analyst. Your task is to analyze data patterns and predict next month's vaccine usage based on the provided daily and monthly vaccine usage data, then compile a complete table and record it in a CSV file.

Daily vaccine usage data:

<daily_vaccine_usage>

A1,1,2014-01-02

A1,1,2014-01-03

A1,1,2014-01-07

...

</daily_vaccine_usage>

Monthly vaccine usage data:

<monthly_vaccine_usage>

A1,20,2014-02

A1,32,2014-03

A1,33,2014-04

...

</monthly_vaccine_usage>

When analyzing the data, consider the following factors:

Temporal factors: Trends, seasonality, and cyclicality in vaccine usage.

Epidemiological factors: Disease transmission dynamics and population immunity levels.

First, perform a detailed analysis of data patterns within <thinking> tags, explaining how you incorporated the above factors.

<thinking>

[Detailed analysis of data patterns here]

</thinking>

Then, provide next month's predicted vaccine usage within <prediction results> tags.

<prediction results>

[Predicted vaccine usage for next month here]

</prediction results>

Next, compile the analysis and predictions into a complete table containing Vaccine Name, Monthly Prediction, and Date. Include the table content within <table content> tags.

<table content>

Vaccine Name, Monthly Prediction, Date

...

</table content>

Finally, save this table to a CSV file named "vaccine_usage_prediction.csv".

(4) Real Demand Prompt Template

You are a vaccine usage data analyst. Your responsibility is to compare actual monthly vaccine usage against model-predicted usage, calculate the error magnitude, and compute system calibration values when necessary.

Below is the actual monthly vaccine usage:

<actual_usage>

Vaccine Name,Total Usage Quantity,Date

A1,20,2014-02

A1,32,2014-03

A1,33,2014-04

...

</actual_usage>

Below is the model-predicted monthly vaccine usage:

<predicted_usage>

Vaccine Name, Monthly Prediction, Date

...

</predicted_usage>

Please conduct the analysis following these steps:

Calculate the ratio of the predicted value to the actual value for each month (Formula: Predicted Value / Actual Value).

Determine if the ratio for each month falls within the acceptable range of 95% ~ 105%.

If a month's ratio falls outside the acceptable range, calculate a corresponding 95% Confidence Interval (CI) Calibration Coefficient such that the Final Adjusted Prediction = Predicted Value * Calibration Coefficient falls within the 95% ~ 105% acceptable range.

Record the ratio and the calibration coefficient (if needed) for each month.

Compute the System Calibration Value (Value Correction).

Within the <thinking> tags, provide a detailed explanation of your calculation process and reasoning. Then, output the following results within the <result> tags:

The ratio of predicted value to actual value for each month.

Whether calibration is needed for each month (Yes/No).

The calibration coefficient for each month (if applicable).

The System Calibration Value (Value Correction).

<thinking>

[Here, detail your calculation process and reasoning.]

</thinking>

<result>

[Here output the monthly ratios, whether calibration is needed, calibration coefficients (if applicable), and the System Calibration Value.]

</result>

(5) Vaccine Prompt Template

You are a vaccine usage data analyst. Your task is to calculate the final monthly vaccine usage based on given monthly predictions and system correction values for the same vaccine, then compile the results into a complete table and record it in a CSV file.

Here is the monthly prediction data:

<monthly_predictions>

Vaccine Name, Monthly Prediction, Date

...

</monthly_predictions>

Here are the system correction values:

<value_correction>

Vaccine Name, Value Correction, Date

...

</value_correction>

The calculation formula is: Final Predicted Value = Predicted Value × Correction Value.

First, explain the calculation process in detail within the <thinking> tag. Then organize the data in the following table format:

Month Predicted Value Correction Value Final Predicted Value

[Month 1] [Predicted Value 1] [Correction Value] [Final Value 1]

[Month 2] [Predicted Value 2] [Correction Value] [Final Value 2]

... ... ... ...

Finally, convert the table into CSV format with comma-separated values and headers in the first row.

Write the CSV content inside the <csv_content> tag.

<thinking>

[Explain the calculation process here]

</thinking>

<csv_content>

[Enter CSV file content here]

</csv_content>
