# Supplementary material for: The adaptive large language models for vaccine prediction: A novel approach to vaccine demand prediction with engineered deviation prompts
Source: PLOS Digit Health. 2026 Mar 9;5(3):e0001273. doi: 10.1371/journal.pdig.0001273 (PMC12970898; doi:10.1371/journal.pdig.0001273)
Supplement: S2 Appendix — (DOCX) [file pdig.0001273.s002.docx]

**Appendix B: Parameter Specifications for Predictive Modeling Approaches**

Logistic Regression (LR):

The logistic regression model was implemented using the scikit-learn library (version 1.7.0). The solver parameter was modified to 'lbfgs', with all other parameters maintained at their default settings; detailed specifications are available in the official scikit-learn documentation at

https://scikit-learn.org/stable/modules/generated/sklearn.linear_model.LogisticRegression.html.

Random Forest (RF):

The random forest model was implemented using the scikit-learn library (version 1.7.0). Specific modifications included setting n_estimators=100 and random_state=42, while all other parameters remained at default values; comprehensive details can be referenced in the official documentation at

https://scikit-learn.org/stable/modules/generated/sklearn.ensemble.RandomForestRegressor.html.

Long Short-Term Memory (LSTM):

This LSTM model architecture comprised a single LSTM layer with four neurons and an output layer with one fully connected neuron, featuring an input shape of (1,1) to represent one time step and one feature for prediction. Key parameters included a time step n=1 for supervised dataset construction, a training batch size of 1, 100 epochs, and an 80:20 train-test split ratio; data were normalized via MinMaxScaler, with training employing mean squared error loss, the Adam optimizer, and denormalized predictions outputted for the target date.

GPT-4 API:

The GPT-4 API was accessed using the 'gpt-4.1' model, with generation parameters set to temperature=0.2, max_tokens=1000, and frequency_penalty=0.0, and the system prompt specified as "You are a helpful assistant".
