# Supplementary material for: The adaptive large language models for vaccine prediction: A novel approach to vaccine demand prediction with engineered deviation prompts
Source: PLOS Digit Health. 2026 Mar 9;5(3):e0001273. doi: 10.1371/journal.pdig.0001273 (PMC12970898; doi:10.1371/journal.pdig.0001273)
Supplement: S3 Appendix — (DOCX) [file pdig.0001273.s003.docx]

**Appendix C: Methodology of the Multi-step Monthly Rolling-Forecast Evaluation**

**C.1. Rationale and Objective**

This appendix details the multi-step monthly rolling-forecast scheme employed for the temporal evaluation of the ALLMVP model. This approach simulates a realistic operational scenario where the model is updated with newly forecasted data on a monthly basis to predict subsequent months, thereby rigorously testing its multi-step forecasting capability in a pseudo-productive environment. The final annual aggregates are derived from the summation of these monthly predictions, aligning with the cumulative nature of annual vaccine procurement planning.

**C.2. Experimental Setup and Data Partitioning**

The evaluation framework is based on a clear temporal split:

• Initial Training Set: Data from January 2014 to December 2017.

• Sequential Testing Periods: Data from January 2018 to December 2022. The model generates forecasts for each year within this period through an iterative, expanding-window process.

**C.3. Step-by-Step Forecasting Procedure**

The core of the methodology involves a monthly roll-forward mechanism within each forecast year, as conceptually illustrated in Figure C1.

**Iteration 1: Forecasting for the Year 2018**

This process begins with the initial training window and proceeds monthly:

1. Forecast January 2018: The ALLMVP model is trained on data from Jan 2014-Dec 2017 and generates a prediction for January 2018.
2. Expand Training Window (Pseudo-Expansion): The predicted value for January 2018 is incorporated into the training dataset. This creates an expanded training set spanning Jan 2014 - Dec 2017 + Predicted Jan 2018.
3. Forecast February 2018: The model is retrained on this expanded set to predict February 2018.
4. Iterate Monthly: Steps 2 and 3 are repeated for each subsequent month (March 2018, April 2018, ..., December 2018). At each step, the training window is expanded to include all previous months of 2018, albeit with their predicted values.
5. Annual Aggregation: After the December 2018 prediction is made, the 12 monthly predicted values for 2018 are summed to produce the final annual forecast for 2018.

**Iteration 2: Forecasting for the Year 2019**

The process continues with a updated baseline:

• The training window is now reset to the actual historical data from January 2014 to December 2018 (which includes the actual values for 2018, not the predicted ones from Iteration 1).

• The entire monthly procedure described in Iteration 1 is repeated to generate monthly forecasts for 2019, which are then summed to produce the annual forecast for 2019.

**Subsequent Iterations:**

This pattern continues—using all available actual data up to the end of the previous year as the baseline for the next year's monthly forecasting cycle—until the entire test period (2018-2022) is covered.

**C.4. Advantages and Operational Rationale**

• Simulates Sequential Decision-Making: This method realistically mimics a scenario where planners must make decisions for the next period based on the best available forecast, even before actual data is confirmed.

• Rigorous Multi-step Test: It represents a challenging and robust test of the model's ability to maintain accuracy over a long forecasting horizon without being frequently corrected with actual data.

• Prevents Data Leakage: The evaluation for any given year uses only data available strictly prior to that year, ensuring a pure out-of-sample test.

**Figure C1: Schematic diagram of the multi-step monthly rolling-forecast procedure**


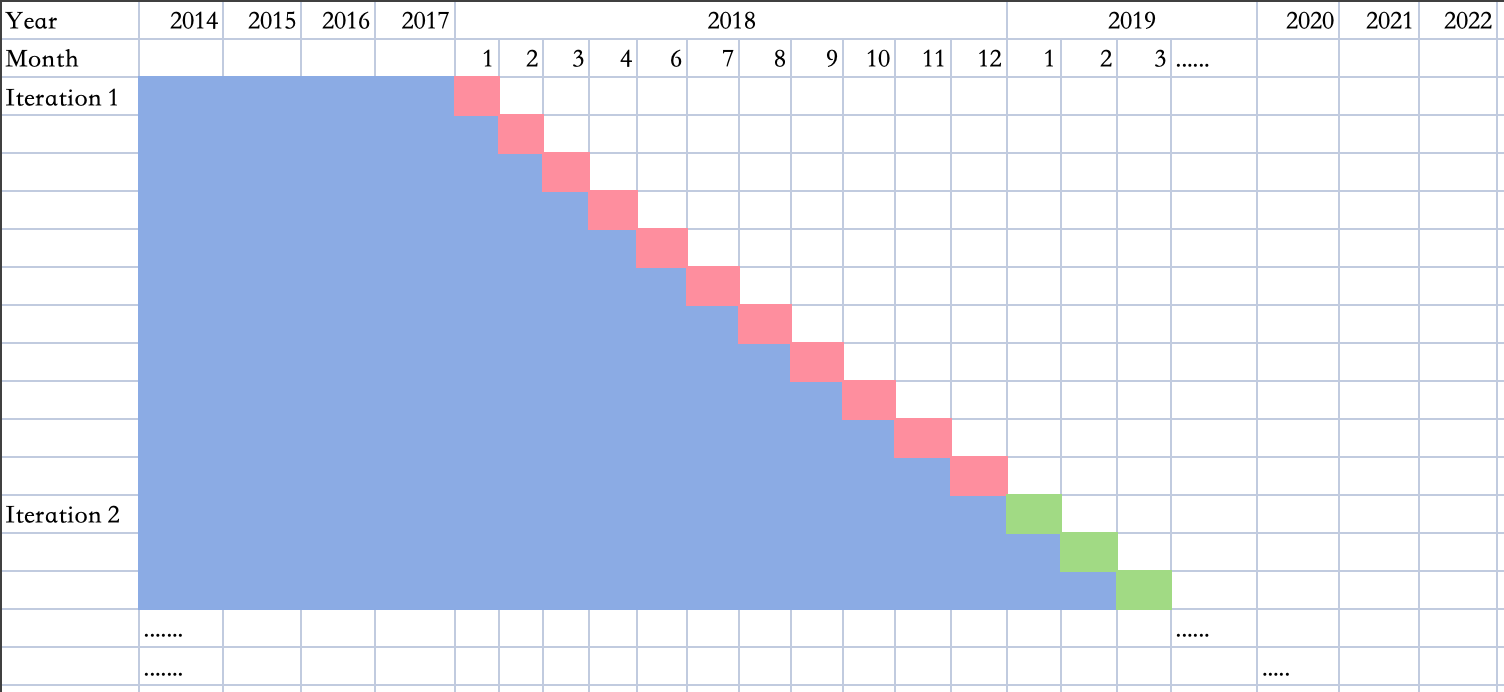


**Table C1: Summary of the Multi-step Rolling-Forecast Evaluation**

| Forecast Year | Baseline Training Data (Actuals) | Forecasting Mechanism | Annual Forecast Derivation |
| --- | --- | --- | --- |
| 2018 | Jan 2014 - Dec 2017 | 12 sequential monthly forecasts, each using pseudo-expanded training data | Sum of 12 monthly predictions |
| 2019 | Jan 2014 - Dec 2018 | 12 sequential monthly forecasts, each using pseudo-expanded training data | Sum of 12 monthly predictions |
| ... | ... | ... | ... |
| 2022 | Jan 2014 - Dec 2021 | 12 sequential monthly forecasts, each using pseudo-expanded training data | Sum of 12 monthly predictions |
