# Supplementary material for: The adaptive large language models for vaccine prediction: A novel approach to vaccine demand prediction with engineered deviation prompts
Source: PLOS Digit Health. 2026 Mar 9;5(3):e0001273. doi: 10.1371/journal.pdig.0001273 (PMC12970898; doi:10.1371/journal.pdig.0001273)
Supplement: S1 Table — (DOCX) [file pdig.0001273.s004.docx]

**Table 1：Comparative predictive value of LLMVP and ALLMVP models from 2018 to 2022**

|  |  | A1 | A3 | A4 | A5 | A6 | A7 | A8 |
| --- | --- | --- | --- | --- | --- | --- | --- | --- |
| 2018 | True | 18907 | 22325 | 12802 | 33231 | 30405 | 20720 | 18765 |
|  | LR | 22153 (1.1717) | 25061 (1.1226) | 16262 (1.2703) | 35033 (1.0542) | 33062 (1.0874) | 20821 (1.0049) | 19581 (1.0435) |
|  | A-LR | 19739 (1.0440) | 23549 (1.0548) | 15622 (1.2203) | 34552 (1.0398) | 32133 (1.0568) | 21145 (1.0205) | 18911 (1.0078) |
|  | RF | 21612 (1.1431) | 23860 (1.0688) | 16193 (1.2649) | 36284 (1.0919) | 31745 (1.0441) | 21360 (1.0309) | 19235 (1.0250) |
|  | A-RF | 19550 (1.0340) | 22955 (1.0282) | 13966 (1.0909) | 34475 (1.0374) | 31116 (1.0234) | 21079 (1.0173) | 18941 (1.0094) |
|  | LSTM | 20824 (1.1014) | 25281 (1.1324) | 16688 (1.3035) | 34647 (1.0426) | 30170 (0.9923) | 20664 (0.9973) | 19129 (1.0194) |
|  | A-LSTM | 19405 (1.0263) | 23123 (1.0357) | 13533 (1.0571) | 34256 (1.0308) | 30975 (1.0187) | 21227 (1.0245) | 19072 (1.0164) |
|  | LLMVP | 22591 (1.1948) | 24749 (1.1086) | 16751 (1.3085) | 40126 (1.2075) | 32585 (1.0717) | 24337 (1.1746) | 19666 (1.0480) |
|  | ALLMVP | 19737 (1.0439) | 23055 (1.0327) | 13496 (1.0542) | 35331 (1.0632) | 30869 (1.0153) | 21216 (1.0239) | 19550 (1.0418) |
| 2019 | True | 17135 | 17400 | 10240 | 31651 | 27227 | 18054 | 15479 |
|  | LR | 19128 (1.1163) | 22631 (1.3006) | 10677 (1.0427) | 36392 (1.1498) | 32348 (1.1881) | 21471 (1.1893) | 19703 (1.2729) |
|  | A-LR | 17268 (1.0078) | 17399 (0.9999) | 9860 (0.9629) | 31622 (0.9991) | 28945 (1.0631) | 18414 (1.0199) | 16291 (1.0525) |
|  | RF | 19751 (1.1527) | 21278 (1.2229) | 10681 (1.0431) | 34079 (1.0767) | 29882 (1.0975) | 18446 (1.0217) | 19715 (1.2737) |
|  | A-RF | 17139 (1.0002) | 17286 (0.9934) | 9908 (0.9676) | 31523 (0.9960) | 28558 (1.0489) | 18122 (1.0038) | 16124 (1.0417) |
|  | LSTM | 19809 (1.1561) | 22369 (1.2856) | 13996 (1.3668) | 33964 (1.0731) | 30275 (1.1119) | 19155 (1.0610) | 18915 (1.2220) |
|  | A-LSTM | 16932 (0.9882) | 17213 (0.9893) | 10155 (0.9917) | 31413 (0.9925) | 27511 (1.0104) | 18071 (1.0009) | 15647 (1.0109) |
|  | LLMVP | 19818 (1.1566) | 21726 (1.2486) | 11836 (1.1559) | 34405 (1.0870) | 31538 (1.1583) | 20870 (1.1560) | 18202 (1.1759) |
|  | ALLMVP | 16743 (0.9771) | 17220 (0.9897) | 10284 (1.0043) | 31740 (1.0028) | 27988 (1.0280) | 17971 (0.9954) | 15908 (1.0277) |
| 2020 | True | 17187 | 20453 | 11503 | 32136 | 27611 | 17234 | 13494 |
|  | LR | 20095 (1.1692) | 24921 (1.2185) | 11454 (0.9957) | 34717 (1.0803) | 31424 (1.1381) | 19187 (1.1133) | 17075 (1.2654) |
|  | A-LR | 17141 (0.9973) | 22664 (1.1081) | 12226 (1.0629) | 41661 (1.2964) | 35327 (1.2795) | 22012 (1.2772) | 18681 (1.3844) |
|  | RF | 19161 (1.1149) | 22588 (1.1044) | 11699 (1.0170) | 34486 (1.0731) | 30487 (1.1042) | 19000 (1.1025) | 15988 (1.1848) |
|  | A-RF | 17109 (0.9955) | 22215 (1.0861) | 12507 (1.0873) | 34641 (1.0779) | 28112 (1.0181) | 18705 (1.0854) | 15633 (1.1585) |
|  | LSTM | 19751 (1.1492) | 23655 (1.1566) | 13944 (1.2122) | 34402 (1.0705) | 30269 (1.0963) | 19038 (1.1047) | 18683 (1.3845) |
|  | A-LSTM | 17393 (1.0120) | 20987 (1.0261) | 11574 (1.0062) | 33608 (1.0458) | 27621 (1.0004) | 18219 (1.0572) | 13511 (1.0013) |
|  | LLMVP | 16839 (0.9798) | 18802 (0.9193) | 9190 (0.7989) | 31521 (0.9809) | 28157 (1.0198) | 17390 (1.0091) | 14467 (1.0721) |
|  | ALLMVP | 17316 (1.0075) | 20497 (1.0022) | 11996 (1.0429) | 33284 (1.0357) | 27215 (0.9857) | 14016 (0.8133) | 13556 (1.0046) |
| 2021 | True | 17078 | 21239 | 5866 | 28925 | 23243 | 14182 | 13659 |
|  | LR | 20320 (1.1898) | 25795 (1.2145) | 10039 (1.7114) | 29232 (1.0106) | 31429 (1.3522) | 14629 (1.0315) | 16716 (1.2238) |
|  | A-LR | 17650 (1.0335) | 23593 (1.1108) | 6863 (1.1700) | 28062 (0.9702) | 29229 (1.2575) | 17631 (1.2432) | 13823 (1.0120) |
|  | RF | 18634 (1.0911) | 22905 (1.0784) | 7657 (1.3053) | 31227 (1.0796) | 30960 (1.3320) | 15698 (1.1069) | 16403 (1.2009) |
|  | A-RF | 17463 (1.0225) | 22600 (1.0641) | 6525 (1.1123) | 29184 (1.0090) | 26071 (1.1217) | 14843 (1.0466) | 13563 (0.9930) |
|  | LSTM | 19359 (1.1336) | 23674 (1.1146) | 8706 (1.4841) | 33140 (1.1457) | 29978 (1.2898) | 17035 (1.2012) | 18606 (1.3622) |
|  | A-LSTM | 17353 (1.0161) | 24887 (1.1718) | 6213 (1.0592) | 29493 (1.0196) | 23514 (1.0117) | 14141 (0.9971) | 13760 (1.0074) |
|  | LLMVP | 16473 (0.9646) | 18355 (0.8642) | 7004 (1.1940) | 30452 (1.0528) | 27334 (1.1760) | 15324 (1.0805) | 13343 (0.9769) |
|  | ALLMVP | 17359 (1.0165) | 22935 (1.0799) | 6386 (1.0886) | 30004 (1.0373) | 23461 (1.0094) | 14016 (0.9883) | 13702 (1.0031) |
| 2022 | True | 16843 | 9014 | 4212 | 28636 | 19032 | 11929 | 10816 |
|  | LR | 18484 (1.0974) | 16079 (1.7838) | 14614 (3.4696) | 33005 (1.1526) | 24635 (1.2944) | 15863 (1.3298) | 11818 (1.0926) |
|  | A-LR | 19541 (1.1602) | 34385 (3.8146) | 14670 (3.4829) | 34750 (1.2135) | 19813 (1.0410) | 66532 (5.5773) | 34389 (3.1795) |
|  | RF | 18942 (1.1246) | 12275 (1.3618) | 5015 (1.1906) | 32272 (1.1270) | 27532 (1.4466) | 15214 (1.2754) | 12666 (1.1710) |
|  | A-RF | 18589 (1.1037) | 22284 (2.4722) | 13783 (3.2723) | 34214 (1.1948) | 26163 (1.3747) | 19981 (1.6750) | 16681 (1.5423) |
|  | LSTM | 18976 (1.1266) | 14659 (1.6262) | 6404 (1.5204) | 32651 (1.1402) | 28507 (1.4978) | 16046 (1.3451) | 16828 (1.5558) |
|  | A-LSTM | 18516 (1.0993) | 11435 (1.2686) | 7018 (1.6662) | 33764 (1.1791) | 20170 (1.0598) | 17228 (1.4442) | 12300 (1.1372) |
|  | LLMVP | 16092 (0.9554) | 16116 (1.7879) | 4219 (1.0017) | 28620 (0.9994) | 23155 (1.2166) | 12980 (1.0881) | 12167 (1.1249) |
|  | ALLMVP | 17349 (1.0300) | 9127 (1.0125) | 3844 (0.9126) | 30280 (1.0574) | 20442 (1.0741) | 12563 (1.0531) | 11506 (1.0638) |

Note: The ratios in parentheses represent the ratios of the algorithm's predicted values to the true values.
