# Supplementary material for: The adaptive large language models for vaccine prediction: A novel approach to vaccine demand prediction with engineered deviation prompts
Source: PLOS Digit Health. 2026 Mar 9;5(3):e0001273. doi: 10.1371/journal.pdig.0001273 (PMC12970898; doi:10.1371/journal.pdig.0001273)
Supplement: S2 Table — (DOCX) [file pdig.0001273.s005.docx]

**Table 2: Comparison of Model Predicted Quantities for Each Vaccine from 2018 to 2022**

|  | A1 | A3 | A4 | A5 | A6 | A7 | A8 |
| --- | --- | --- | --- | --- | --- | --- | --- |
| True | 87150 | 90431 | 44623 | 154579 | 127518 | 82119 | 72213 |
| LR | 100180 (1.1495) | 114487 (1.2660) | 63046 (1.4129) | 168379 (1.0893) | 152898 (1.1990) | 91971 (1.1200) | 84893 (1.1756) |
| A-LR | 91339 (1.0481) | 121590 (1.3446) | 59241 (1.3276) | 170647 (1.1039) | 145447 (1.1406) | 145734 (1.7747) | 102095 (1.4138) |
| RF | 98100 (1.1256) | 102906 (1.1380) | 51245 (1.1484) | 168348 (1.0891) | 150606 (1.1811) | 89718 (1.0925) | 84007 (1.1633) |
| A-RF | 89850 (1.0310) | 107340 (1.1870) | 56689 (1.2704) | 164037 (1.0612) | 140020 (1.0980) | 92730 (1.1292) | 80942 (1.1209) |
| LSTM | 98719 (1.1327) | 109638 (1.2124) | 59738 (1.3387) | 168804 (1.0920) | 149199 (1.1700) | 91938 (1.1196) | 92161 (1.2762) |
| A-LSTM | 89599 (1.0281) | 97645 (1.0798) | 48493 (1.0867) | 162534 (1.0515) | 129791 (1.0178) | 88886 (1.0824) | 74290 (1.0288) |
| LLMVP | 91813 (1.0535) | 99748 (1.1030) | 49000 (1.0981) | 165124 (1.0682) | 142769 (1.1196) | 90901 (1.1069) | 77845 (1.0780) |
| ALLMVP | 88504 (1.0155) | 92834 (1.0266) | 46006 (1.0310) | 160639 (1.0392) | 129975 (1.0193) | 79782 (0.9715) | 74222 (1.0278) |
