# Supplementary material for: The adaptive large language models for vaccine prediction: A novel approach to vaccine demand prediction with engineered deviation prompts
Source: PLOS Digit Health. 2026 Mar 9;5(3):e0001273. doi: 10.1371/journal.pdig.0001273 (PMC12970898; doi:10.1371/journal.pdig.0001273)
Supplement: S3 Table — (DOCX) [file pdig.0001273.s006.docx]

**Table 3: Comparison of Model Predicted Total Vaccine Quantities from 2018 to 2022**

|  | 2018 | 2019 | 2020 | 2021 | 2022 |
| --- | --- | --- | --- | --- | --- |
| True | 157155 | 137186 | 139618 | 124192 | 100482 |
| LR | 171973 (1.0943) | 162350 (1.1834) | 158873 (1.1379) | 148160 (1.1930) | 134498 (1.3385) |
| A-LR | 165651 (1.0541) | 139799 (1.0190) | 169712 (1.2155) | 136851 (1.1019) | 224080 (2.2301) |
| RF | 170289 (1.0836) | 153832 (1.1213) | 153409 (1.0988) | 143484 (1.1553) | 123916 (1.2332) |
| A-RF | 162082 (1.0314) | 138660 (1.0107) | 148922 (1.0666) | 130249 (1.0488) | 151695 (1.5097) |
| LSTM | 167403 (1.0652) | 158483 (1.1552) | 159742 (1.1441) | 150498 (1.2118) | 134071 (1.3343) |
| A-LSTM | 161591 (1.0282) | 136942 (0.9982) | 142913 (1.0236) | 129361 (1.0416) | 120431 (1.1985) |
| LLMVP | 180805 (1.1505) | 158395 (1.1546) | 136366 (0.9767) | 128285 (1.0330) | 113349 (1.1281) |
| ALLMVP | 163254 (1.0388) | 137854 (1.0049) | 137880 (0.9876) | 127863 (1.0296) | 105111 (1.0461) |
