# Supplementary material for: The adaptive large language models for vaccine prediction: A novel approach to vaccine demand prediction with engineered deviation prompts
Source: PLOS Digit Health. 2026 Mar 9;5(3):e0001273. doi: 10.1371/journal.pdig.0001273 (PMC12970898; doi:10.1371/journal.pdig.0001273)
Supplement: S4 Table — (DOCX) [file pdig.0001273.s007.docx]

**Table 4: Summary of forecast error distributions by vaccine type (2018-2022)**

(a) Year-by-Year Forecast and Error Values

|  | Year | True Demand (doses) | Predicted Demand (doses) | Absolute Error | Percentage Error (%) | Bias |
| --- | --- | --- | --- | --- | --- | --- |
| A1 | 2018 | 18907 | 19737 | 830 | 4.39 | 830 |
|  | 2019 | 17135 | 16743 | 392 | 2.29 | -392 |
|  | 2020 | 17187 | 17316 | 129 | 0.75 | 129 |
|  | 2021 | 17078 | 17359 | 281 | 1.65 | 281 |
|  | 2022 | 16843 | 17349 | 506 | 3.0 | 506 |
| A3 | 2018 | 22325 | 23055 | 730 | 3.27 | 730 |
|  | 2019 | 17400 | 17220 | 180 | 1.03 | -180 |
|  | 2020 | 20453 | 20497 | 44 | 0.22 | 44 |
|  | 2021 | 21239 | 22935 | 1696 | 7.99 | 1696 |
|  | 2022 | 9014 | 9127 | 113 | 1.25 | 113 |
| A4 | 2018 | 12802 | 13496 | 694 | 5.42 | 694 |
|  | 2019 | 10240 | 10284 | 44 | 0.43 | 44 |
|  | 2020 | 11503 | 11996 | 493 | 4.29 | 493 |
|  | 2021 | 5866 | 6386 | 520 | 8.86 | 520 |
|  | 2022 | 4212 | 3844 | 368 | 8.74 | -368 |
| A5 | 2018 | 33231 | 35331 | 2100 | 6.32 | 2100 |
|  | 2019 | 31651 | 31740 | 89 | 0.28 | 89 |
|  | 2020 | 32136 | 33284 | 1148 | 3.57 | 1148 |
|  | 2021 | 28925 | 30004 | 1079 | 3.73 | 1079 |
|  | 2022 | 28636 | 30280 | 1644 | 5.74 | 1644 |
| A6 | 2018 | 30405 | 30869 | 464 | 1.53 | 464 |
|  | 2019 | 27227 | 27988 | 761 | 2.8 | 761 |
|  | 2020 | 27611 | 27215 | 396 | 1.43 | -396 |
|  | 2021 | 23243 | 23461 | 218 | 0.94 | 218 |
|  | 2022 | 19032 | 20442 | 1410 | 7.41 | 1410 |
| A7 | 2018 | 20720 | 21216 | 496 | 2.39 | 496 |
|  | 2019 | 18054 | 17971 | 83 | 0.46 | -83 |
|  | 2020 | 17234 | 14016 | 3218 | 18.67 | -3218 |
|  | 2021 | 14182 | 14016 | 166 | 1.17 | -166 |
|  | 2022 | 11929 | 12563 | 634 | 5.31 | 634 |
| A8 | 2018 | 18765 | 19550 | 785 | 4.18 | 785 |
|  | 2019 | 15479 | 15908 | 429 | 2.77 | 429 |
|  | 2020 | 13494 | 13556 | 62 | 0.46 | 62 |
|  | 2021 | 13659 | 13702 | 43 | 0.31 | 43 |
|  | 2022 | 10816 | 11506 | 690 | 6.38 | 690 |

(b) Aggregate Forecast Error Metrics (2018-2022)

| Vaccine Type | Sample Size (years) | MAE (doses) | MAPE (%) | MBE (doses) | Analysis of Systematic Bias |
| --- | --- | --- | --- | --- | --- |
| A1 | 5 | 427.6 | 2.416 | 270.8 | Significant over-forecasting- Consistent over-prediction by ~271 doses on average |
| A3 | 5 | 552.6 | 2.752 | 480.6 | Pronounced over-forecasting- Strongest bias among all vaccines |
| A4 | 5 | 423.8 | 5.548 | 276.6 | Clear over-forecasting- Higher MAPE suggests greater relative error |
| A5 | 5 | 1212 | 3.928 | 1212 | Perfect systematic over-forecasting- MBE = MAE indicates all errors are over-predictions |
| A6 | 5 | 649.8 | 2.822 | 491.4 | Substantial over-forecasting- Second strongest positive bias |
| A7 | 5 | 919.4 | 5.6 | -467.4 | Systematic under-forecasting- Only vaccine showing consistent under-prediction |
| A8 | 5 | 401.8 | 2.82 | 401.8 | Perfect systematic over-forecasting- All predictions are over-estimates |

Notes: MAE: Mean Absolute Error; MAPE: Mean Absolute Percentage Error; MBE: Mean Bias Error;

**Analysis of Systematic Bias**

The analysis of Mean Bias Error (MBE) reveals distinct systematic forecasting patterns across different vaccine types, which has direct implications for inventory management planning. The results can be categorized into three groups:

Pronounced Systematic Over-forecasting (MBE > 0):The majority of vaccines (A1, A3, A4, A5, A6, A8) exhibit significant positive MBE values. This indicates a consistent tendency of the model to over-predictdemand. Notably, for vaccines A5 and A8, the MBE is equal to their MAE (e.g., A5 MBE = 1,212; MAE = 1,212), which implies that for every forecast period, the predicted value was higherthan the true value by approximately 1,212 doses. This strong over-forecasting bias suggests that procurement planners could potentially reduce buffer stocksfor these vaccines to minimize wastage and cost, as the model already builds in a substantial upward buffer.

Systematic Under-forecasting (MBE < 0):In contrast, vaccine A7 demonstrates a clear negative MBE (-467.4), signifying a consistent under-predictionof demand. This is a critical finding from a risk management perspective. Under-forecasting poses a higher operational risk than over-forecasting, as it can directly lead to vaccine shortages. For vaccine A7, it is advisable to add a safety bufferto the model's predictions to mitigate the risk of stockouts.

Relationship between MAE and MBE:The closeness of the MAE and MBE values for several vaccines (especially A5 and A8) further confirms that the forecast error is not random but is predominantly driven by a consistent directional bias. The lower MAPE values for most vaccines (e.g., 2.416% for A1) indicate that the absolute size of the error is relatively small compared to the demand volume, yet the systematic nature of the bias is a key insight for optimizing inventory levels.

In summary, the model exhibits a generally conservative bias, predominantly over-forecasting demand. However, the notable exception of vaccine A7 requires a targeted strategy to prevent shortages. These findings enable program managers to implement differentiated inventory policies rather than a one-size-fits-all approach.
