# Supplementary figures and images for: The adaptive large language models for vaccine prediction: A novel approach to vaccine demand prediction with engineered deviation prompts
Source: PLOS Digit Health. 2026 Mar 9;5(3):e0001273. doi: 10.1371/journal.pdig.0001273 (PMC12970898; doi:10.1371/journal.pdig.0001273)

**Appendix Figure 1: The conceptual architecture of A-LR model**


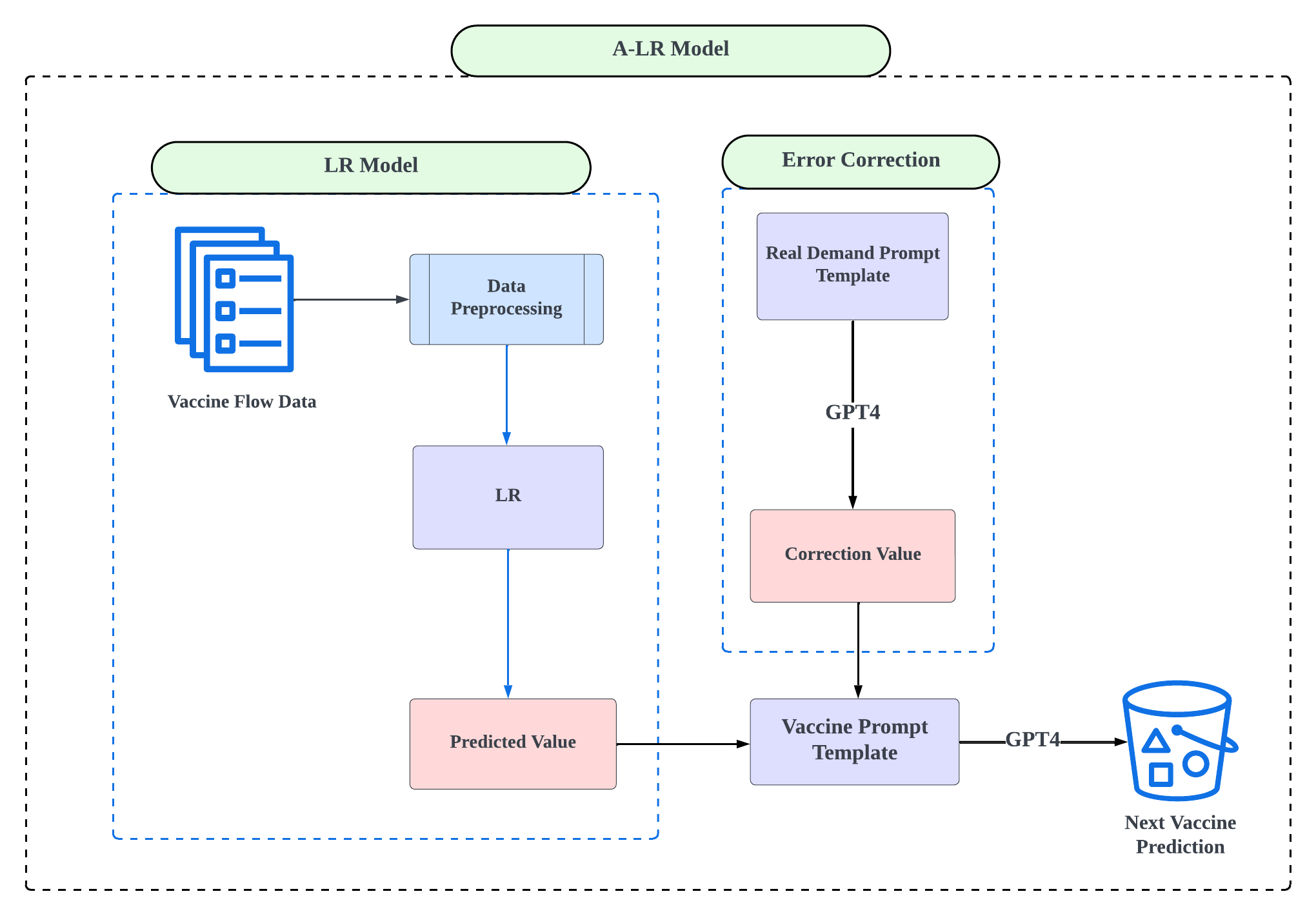

Supplement: S1 Fig — (DOCX) [file pdig.0001273.s008.docx]

**Appendix Figure 2: The conceptual architecture of A-RF model**


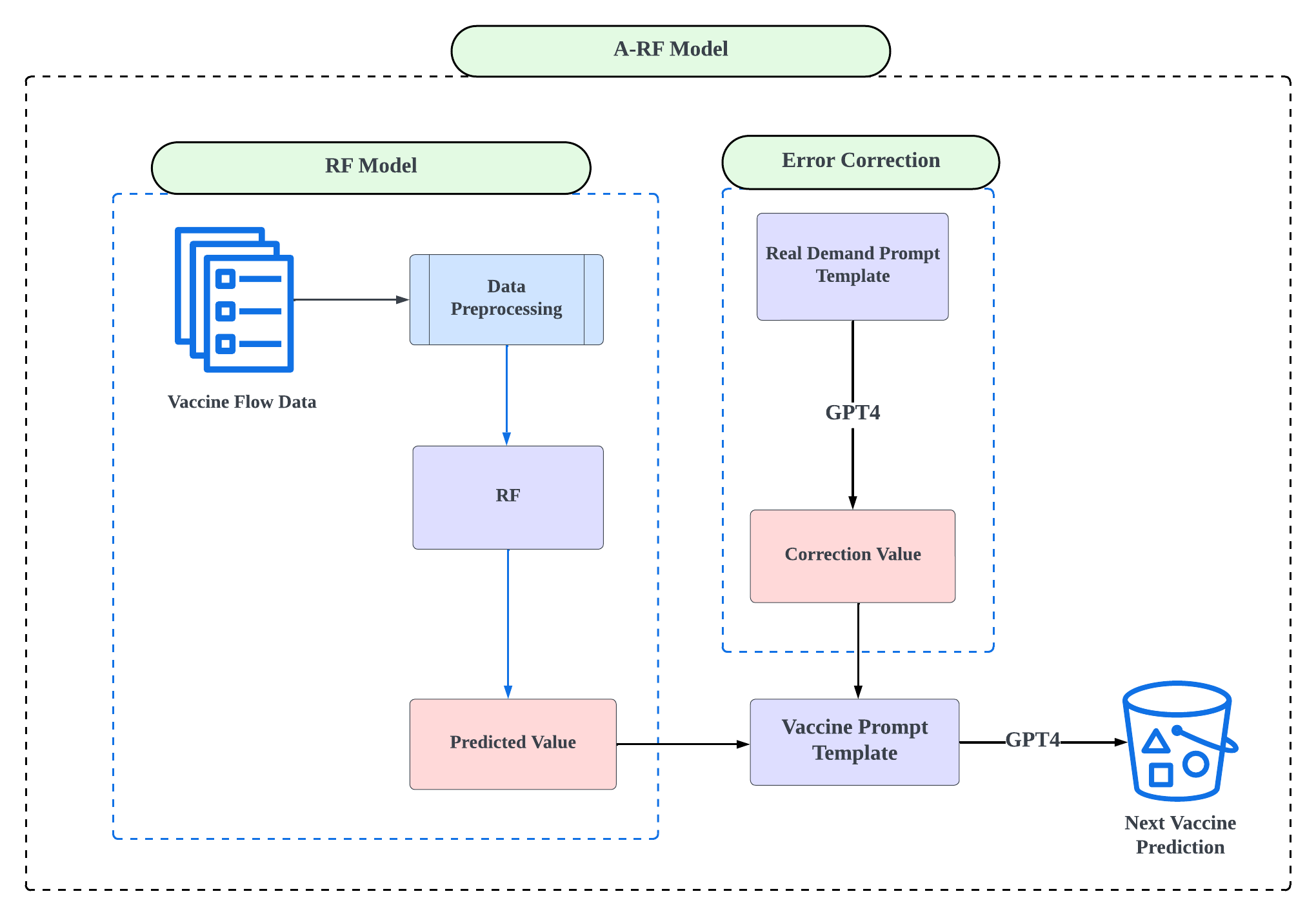

Supplement: S2 Fig — (DOCX) [file pdig.0001273.s009.docx]

**Appendix Figure 3: The conceptual architecture of A-LSTM model**


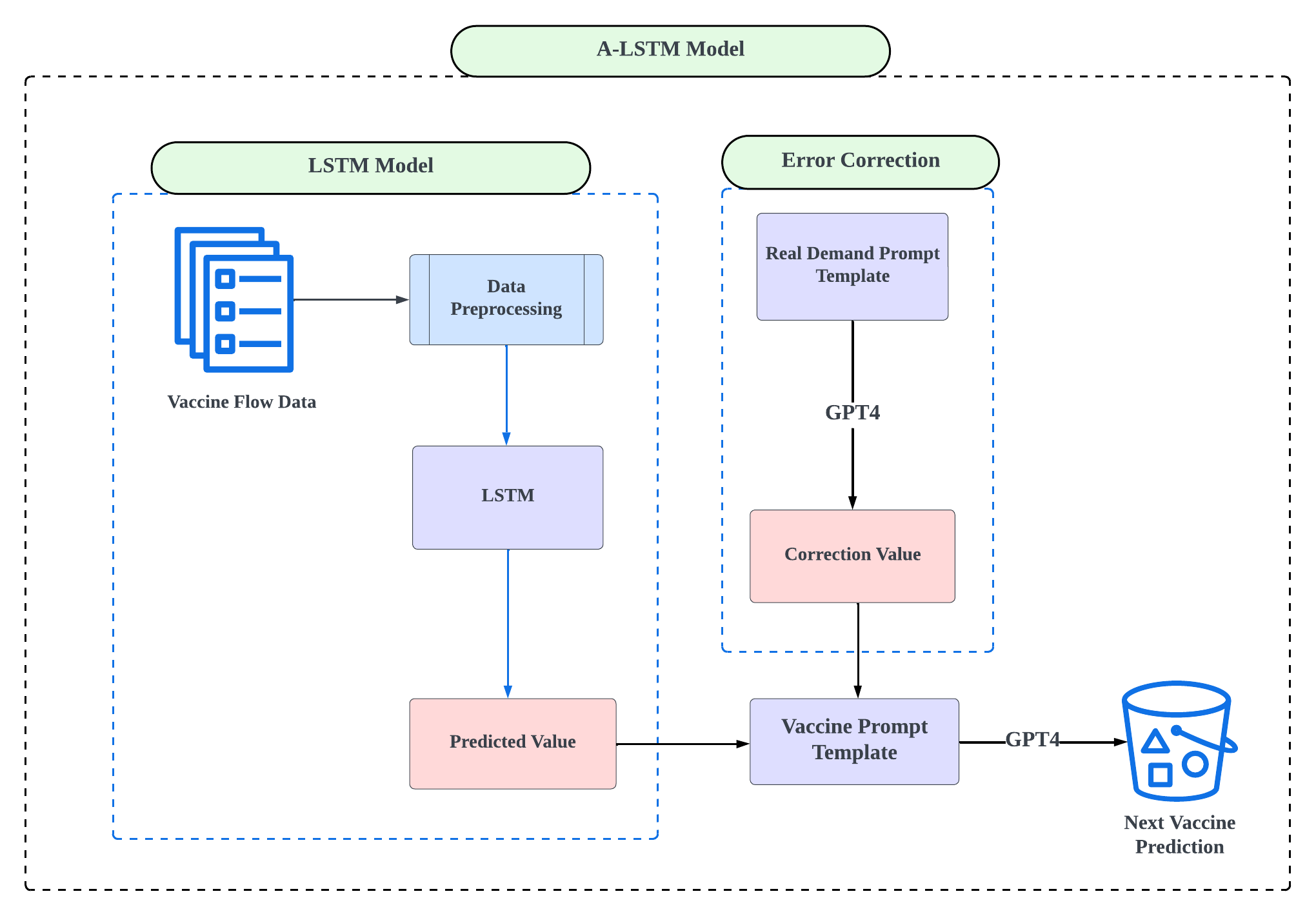

Supplement: S3 Fig — (DOCX) [file pdig.0001273.s010.docx]
