# Supplementary material for: The adaptive large language models for vaccine prediction: A novel approach to vaccine demand prediction with engineered deviation prompts
Source: PLOS Digit Health. 2026 Mar 9;5(3):e0001273. doi: 10.1371/journal.pdig.0001273 (PMC12970898; doi:10.1371/journal.pdig.0001273)
Supplement: Attachment — Submitted filename: Response to Reviewers.docx [file pdig.0001273.s012.docx]

Dear Editor,

We are grateful for the opportunity to revise our paper entitled “**The adaptive large language models for vaccine prediction: A novel approach to vaccine demand prediction with engineered deviation prompts**”. We sincerely appreciate the insightful comments provided by the reviewers, which have significantly strengthened our manuscript. In response, we have thoroughly addressed each point in the accompanying response letter and have made substantial revisions to the paper.

We believe that these enhancements have improved the quality of our work, and we hope the revised manuscript is now suitable for publication in **PLOS Digital Health**. Thank you for your consideration and continued interest in our research.

Please let us know if there are any further questions or if additional information is needed.

Sincerely,

Dr. Tenglong Li

Assistant Professor in Biostatistics

Academy of Pharmacy

Xi’an Jiaotong-Liverpool University

Office: PB246; Phone: 0512-85186479

**Editor Comments:**

1-Please mention in the discussion how: "statistical testing could be used to demonstrate statistical significance in the performance of LLM models against traditional models, as well as adaptive value-correlated models against their non-adaptive counterpart models. Parameter estimation techniques could also be used to better estimate and generalize the predictive strength of the models." This was a suggestion by a reviewer but is NOT required to for publication. Please include it in the discussion on alternate approaches or future research.

*Response: We thank the editor for the constructive feedback, and we have adopted and incorporated the suggestion into the manuscript accordingly.*

*We thank the reviewer for this insightful and critical comment regarding the comparative analysis of model performance. We agree that incorporating statistical significance tests and parameter estimation techniques would provide a more objective and rigorous foundation for comparing the models, and we acknowledge this as a limitation of our current study.*

*In direct response to this valuable suggestion, we have explicitly discussed this point in the "Limitations and Future Work" section of the manuscript (*lines 513-520*). In these lines, we state that while our evaluation presents strong empirical evidence, future work should include statistical testing to formally establish significance between model classes (e.g., LLM-based vs. traditional models) and employ parameter estimation techniques to better generalize the predictive strength of the approaches.*

*We sincerely appreciate the reviewer's guidance, which has not only improved the self-critical awareness of our present manuscript but also provides a clear and valuable direction for our subsequent research.*

**lines 513-520:**

**Statistical Validation: While the comparative analysis of acceptance and deviation ratios provides substantial evidence of performance differences, the incorporation of formal statistical testing (e.g., to demonstrate the significance of differences between LLM-based and traditional models, or between adaptive and non-adaptive counterparts) and parameter estimation techniques (e.g., to compute confidence intervals for accuracy metrics) would further strengthen the validity and generalizability of the conclusions. Implementing such rigorous statistical analyses represents a valuable direction for future work to provide an even more robust evaluation of the model's predictive strength.**

2-Please include a more detailed discussion and literature review on the strength of LLM models in Section 1 could be given in order to provide a more developed reasoning behind the stronger performance of the LLM models against the traditional models as validated in Section 3.

*Response: We appreciate the editor's constructive feedback and have accordingly incorporated this valuable suggestion into our manuscript.*

*We thank the reviewer for this excellent suggestion, which helps to strengthen the theoretical rationale behind our work. We agree that providing a more developed reasoning in the introduction is crucial for contextualizing the empirical results presented in Section 3.*

*In response, we have enriched the literature review and discussion in the Introduction (*Lines 141-149*) to elaborate on the inherent strengths of LLMs that justify their stronger performance. Specifically, we have added a discussion highlighting:*

*（1）The powerful temporal pattern recognition and extrapolation capabilities of LLMs, attributing this to core architectural components like the self-attention mechanism that effectively capture long-range dependencies in data.*

*（2）The exceptional few-shot learning ability of LLMs, facilitated by their prompt-based framework, which allows them to integrate complex information and adapt to limited-data scenarios effectively.*

*We believe these additions have significantly strengthened the logical flow of our manuscript by establishing a clear a priorirationale for the superior performance of our LLM-based approach, which is subsequently validated by the experimental results.*

**lines 141-149:**

**To address these challenges, our study employs large language models (LLMs) for vaccine prediction, motivated by their inherent advantages in temporal analysis. Unlike conventional models, the core architecture of LLMs, such as self-attention mechanisms, enables more flexible capture of long-range dependencies within the data. Furthermore, their strong few-shot learning capabilities and prompt-based framework allow for effective integration of complex information and adaptation to limited data scenarios. These theoretical strengths position LLMs to achieve significantly superior performance compared to traditional methods**

3-Define acronyms on first use; standardize vaccine names across text/tables.

*Response: We thank the reviewer for this important reminder regarding academic rigor and presentation clarity. We sincerely apologize for these oversights in our original manuscript.*

*In response, we have thoroughly reviewed the entire manuscript to ensure that all acronyms are defined upon their first use. Furthermore, we have standardized the names of all vaccines across the main text, tables, and appendices to eliminate any inconsistency. Additionally, we have included a list of abbreviations (*lines 544-559*) for the readers' convenience.*

*We believe these efforts have significantly improved the readability and professionalism of our manuscript.*

**lines 544-559:**

**List of Abbreviations:**

**A-LR: Logistic Regression with LLM Correction**

**A-RF: Random Forest with LLM Correction**

**A-LSTM: LSTM with LLM Correction**

**AI: Artificial Intelligence**

**ALLMVP: Adaptive Large Language Model for Vaccine Prediction**

**LLM: Large Language Model**

**LLMVP: Large Language Model for Vaccine Prediction**

**LR: Logistic Regression**

**LSTM: Long Short-Term Memory**

**MMR: Measles-Mumps-Rubella**

**NIP: National Immunization Program**

**RF: Random Forest**

**RMSE: Root Mean Square Error**

**SIPIMS: Shanghai Immunization Program Information Management System**

**WHO: World Health Organization**

4-Improve some figure captions (axes, units) and consider adding prediction intervals or error bars where appropriate

*Response: We are grateful to the reviewer for this highly insightful suggestion, which significantly contributes to enhancing the clarity and professionalism of our manuscript. The reviewer's expertise in data presentation is greatly appreciated.*

*In direct response to this valuable comment, we have thoroughly reviewed and optimized all figure captions throughout the manuscript. The improvements are focused on making the captions more descriptive and reader-friendly, ensuring they effectively guide the interpretation of the figures.*

*We sincerely thank the reviewer for this constructive feedback, which has undoubtedly improved the overall quality of our work.*

**Reviewer Comments:**

**Reviewer 1**

Using records from 2014–2022 across seven National Immunization Program (NIP) vaccines in Xuhui District (Shanghai), the study reports improved alignment with realized annual demand and an “acceptance ratio” of 71.43% relative to several comparators. The topic reducing wastage and avoiding stockouts via better annual planning is also timely and meaningful for immunization logistics thus this work is promising and practically relevant. The paper is clearly written, the problem is well motivated, and the multi-vaccine, multi-year framing (including post-COVID) is valuable. However, my suggestions below are light-touch and aimed at improving transparency and reproducibility without asking for extensive new experimentation.

Suggestions for improvement (minor, actionable)

1) A short example (one vaccine, one year) or pseudocode showing: inputs → prompt template → LLM output → threshold check → adaptive correction will greatly aid reader understanding. Please also list key LLM settings (model/version, temperature, max tokens/top-p) so others can reproduce the behavior.

*Response: We thank the reviewer for this insightful and helpful suggestion. In response, we have added a short example demonstrating the complete workflow for a specific vaccine and year to enhance readability and reproducibility.*

*Specifically, we have included a concrete code snippet (for one vaccine and one year), illustrating the pipeline from inputs, through the prompt template, to the LLM output, and finally the threshold check and adaptive correction.*

*Furthermore, as suggested, we have provided the corresponding pseudocode and listed the key LLM settings (including the model/version, temperature, and max tokens/top-p parameters) in* **lines 193** *of the manuscript. This includes the specific prompts used to ensure other researchers can reproduce our behavior.*

*We believe these additions greatly aid in reader understanding and facilitate the reproducibility of our method.*

**code snippet, a short example (one vaccine, one year)**

*===================================================================*

*Vaccine_Flow_Data = {*

*"vaccine_A" = {"2014-01-02": 2, "2014-01-02": 1, "2014-01-02": 2, "2014-01-03": 2, "2014-01-04": 4, ...}*

*}*

*for vaccine_name, vaccine_value in Vaccine_Flow_Data:*

*# eg. vaccine_name = vaccine_A, vaccine_value = {"2014-01-02": 2, "2014-01-02": 1, "2014-01-02": 2, "2014-01-03": 2, "2014-01-04": 4, ...}*

*For each month in range 2018 to 2022:*

*vaccine_daily_summary = CALL GPT-4_API(*

*prompt = daily summary prompt,*

*input_data = vaccine_value*

*)*

*# Output: vaccine_daily_summary = {"2014-01-02": 5, "2014-01-03": 2, "2014-01-04": 4, ...} Vaccines on the same date as 2014-01-02 have been accumulated.*

*vaccine_monthly_summary = CALL GPT-4_API(*

*prompt = monthly summary promt,*

*input_data = vaccine_value)*

*# Output: vaccine_montyly_summary = {"2014-01": 11, ...} Vaccines from the same month of 2014-01 have been accumulated.*

*predicted_value = CALL GPT-4_API(*

*prompt = times series prediction prompt,*

*input_data = vaccine_daily_summary, vaccine_monthly_summary)*

*# Output: predicted_value = {'2018-01': 34}*

*correction_value = CALL GPT-4_API(*

*prompt = real demand prompt,*

*input_data = predicted_value, true_value, history_value)*

*# Output: correction_value = {'2018-01': 0.7}*

*final_data_value = CALL GPT-4_API(*

*prompt = vaccine prompt,*

*input_data = predicted value, correction value)*

*# Output: {"month": "2018-01", "predicted_value": 34, "final_predicted_value": 3434, "correction_value": 0.7},*

*PRINT formatted_output FOR vaccine_name:*

*Month, Predicted_Value, Final_Predicted_Value, Correction_Value*

*2018-01, 34, 34, 0.7*

*2018-02, 44, 31, 1.03*

*...*

*===================================================================*

**lines 193:**

**See Appendix A for the pseudo-code.**

**Appendix:**

**Appendix A: Prompt Engineering Framework for Vaccine Demand Prediction**

**This section provides the full pseudo-code for the ALLMVP model.**

**================================================================**

**# PSEUDOCODE: ADAPTIVE LARGE LANGUAGE MODEL FOR VACCINE PREDICTION (ALLMVP)**

**# Step 1: Data Acquisition and Preprocessing**

**Load raw vaccine distribution data from multiple clinics. Preprocess data to generate clean structured format:**

**Vaccine_Flow_Data = {**

**vaccine_1: {'date_day': dose_count, ...} # date_day format: YYYY-MM-DD. Please note that dates may be repeated because the data comes from different clinics, so the same vaccine may be administered at multiple locations on the same day.**

**vaccine_2: {'date_day': dose_count, ...} # dose_count represents the amount of vaccine used that day, for example, 5**

**vaccine_3: {'date_day': dose_count, ...}**

**...**

**}**

**# Step 2: Process Each Vaccine Type**

**FOR each vaccine_name, daily_data in Vaccine_Flow_Data: # eg. vaccine_name = A1, vaccine_value = Vaccine_Flow_Data['A1'] = {'date_day': vaccine_num, ...}**

**For each month in range 2018 to 2022:**

**# 2.1 Generate Daily Summary (Consolidate Multi-Clinic Data)**

**vaccine_daily_summary = CALL GPT-4_API(**

**prompt = daily summary prompt,**

**input_data = vaccine_value**

**)**

**# Output: {"2014-01-01": total_doses, ...} # Unique dates**

**# 2.2 Generate Monthly Aggregation**

**vaccine_monthly_summary = CALL GPT-4_API(**

**prompt = monthly summary promt,**

**input_data = vaccine_value**

**)**

**# Output: {"2014-01": monthly_total, ...}**

**# 2.3 Model Forecasting**

**predicted_value = CALL GPT-4_API(**

**prompt = times series prediction prompt,**

**input_data = vaccine_daily_summary, vaccine_monthly_summary**

**)**

**# Output: {"2018-01": predicted_doses}**

**# 2.4 Demand Correction (Calibration Step)**

**correction_value = CALL GPT-4_API(**

**prompt = real demand prompt,**

**input_data = predicted_value, true_value, history_value**

**)**

**# Output: {"2018-01": correction_ratio}**

**# 2.5 Generate Final Output**

**final_data_value = CALL GPT-4_API(**

**prompt = vaccine prompt,**

**input_data = predicted value, correction value**

**)**

**# Output: {"month": "2018-01", "predicted_value": predicted_value, "final_predicted_value": final_predicted_value, "correction_value": correction_value}**

**# Step 3: Output Results**

**PRINT formatted_output FOR vaccine_name:**

**Month, Predicted_Value, Final_Predicted_Value, Correction_Value**

**2018-01, 1525, 1525, 0.8**

**2018-02, 1676, 1341, 0.49**

**...**

**================================================================**

**The implementation deploys model="gpt-4.1" with controlled generation parameters (temperature=0.2, max_tokens=1000, frequency_penalty=0.0), system prompts is "You are a helpful assistant for text processing", the formal definition of user prompts is as follows:**

**(1) Daily Summary Prompt Template**

**As a vaccine usage data analyst, your task is to process raw daily vaccine usage records. Clean and merge the data, combining records for the same vaccine on the same day into a single total. Finally, organize the data into a complete table and save it to a CSV file.**

**Raw daily vaccine usage records:**

**<Vaccine Usage Raw Records>**

**A1,1,2014-01-02**

**A1,1,2014-01-03**

**A1,1,2014-01-07**

**....**

**</Vaccine Usage Raw Records>**

**Process the data according to the following steps:**

**Carefully inspect the raw records to remove any empty rows, duplicate entries, or obviously erroneous data.**

**Iterate through all records. For entries corresponding to the same vaccine on the same day, sum and combine the usage quantities.**

**Organize the merged data into a complete table with three columns: Vaccine Name, Total Usage Quantity, and Date.**

**After processing, save the table data to a CSV file. Use commas as delimiters. The first line should be the header (Vaccine Name,Total Usage Quantity,Date). Subsequent lines should contain each merged data entry.**

**Write the processed table data within the <Processed Table> tags. Use plain text format with comma-separated columns and newline-separated rows. The first row must be the header.**

**<Processed Table>**

**Vaccine Name,Total Usage Quantity,Date**

**A1,10,2014-01-02**

**A1,12,2014-01-03**

**A1,13,2014-01-07**

**....**

**</Processed Table>**

**(2) Monthly Summary Prompt Template**

**As a vaccine usage data analyst, your task is to process raw daily vaccine usage records. Clean and merge the data, combining records for the same vaccine on the same month into a single total. Finally, organize the data into a complete table and save it to a CSV file.**

**Raw daily vaccine usage records:**

**<Vaccine Usage Raw Records>**

**A1,1,2014-01-02**

**A1,1,2014-01-03**

**A1,1,2014-01-07**

**....**

**</Vaccine Usage Raw Records>**

**Process the data according to the following steps:**

**Carefully inspect the raw records to remove any empty rows, duplicate entries, or obviously erroneous data.**

**Iterate through all records. For entries corresponding to the same vaccine on the same month, sum and combine the usage quantities.**

**Organize the merged data into a complete table with three columns: Vaccine Name, Total Usage Quantity, and Date.**

**After processing, save the table data to a CSV file. Use commas as delimiters. The first line should be the header (Vaccine Name,Total Usage Quantity,Date). Subsequent lines should contain each merged data entry.**

**Write the processed table data within the <Processed Table> tags. Use plain text format with comma-separated columns and newline-separated rows. The first row must be the header.**

**<Processed Table>**

**Vaccine Name,Total Usage Quantity,Date**

**A1,20,2014-02**

**A1,32,2014-03**

**A1,33,2014-04**

**....**

**</Processed Table>**

**(3) Time Series Prediction Prompt Template**

**You are a vaccine usage data analyst. Your task is to analyze data patterns and predict next month's vaccine usage based on the provided daily and monthly vaccine usage data, then compile a complete table and record it in a CSV file.**

**Daily vaccine usage data:**

**<daily_vaccine_usage>**

**A1,1,2014-01-02**

**A1,1,2014-01-03**

**A1,1,2014-01-07**

**...**

**</daily_vaccine_usage>**

**Monthly vaccine usage data:**

**<monthly_vaccine_usage>**

**A1,20,2014-02**

**A1,32,2014-03**

**A1,33,2014-04**

**...**

**</monthly_vaccine_usage>**

**When analyzing the data, consider the following factors:**

**Temporal factors: Trends, seasonality, and cyclicality in vaccine usage.**

**Epidemiological factors: Disease transmission dynamics and population immunity levels.**

**First, perform a detailed analysis of data patterns within <thinking> tags, explaining how you incorporated the above factors.**

**<thinking>**

**[Detailed analysis of data patterns here]**

**</thinking>**

**Then, provide next month's predicted vaccine usage within <prediction results> tags.**

**<prediction results>**

**[Predicted vaccine usage for next month here]**

**</prediction results>**

**Next, compile the analysis and predictions into a complete table containing Vaccine Name, Monthly Prediction, and Date. Include the table content within <table content> tags.**

**<table content>**

**Vaccine Name, Monthly Prediction, Date**

**...**

**</table content>**

**Finally, save this table to a CSV file named "vaccine_usage_prediction.csv".**

**(4) Real Demand Prompt Template**

**You are a vaccine usage data analyst. Your responsibility is to compare actual monthly vaccine usage against model-predicted usage, calculate the error magnitude, and compute system calibration values when necessary.**

**Below is the actual monthly vaccine usage:**

**<actual_usage>**

**Vaccine Name,Total Usage Quantity,Date**

**A1,20,2014-02**

**A1,32,2014-03**

**A1,33,2014-04**

**...**

**</actual_usage>**

**Below is the model-predicted monthly vaccine usage:**

**<predicted_usage>**

**Vaccine Name, Monthly Prediction, Date**

**...**

**</predicted_usage>**

**Please conduct the analysis following these steps:**

**Calculate the ratio of the predicted value to the actual value for each month (Formula: Predicted Value / Actual Value).**

**Determine if the ratio for each month falls within the acceptable range of 95% ~ 105%.**

**If a month's ratio falls outside the acceptable range, calculate a corresponding 95% Confidence Interval (CI) Calibration Coefficient such that the Final Adjusted Prediction = Predicted Value * Calibration Coefficient falls within the 95% ~ 105% acceptable range.**

**Record the ratio and the calibration coefficient (if needed) for each month.**

**Compute the System Calibration Value (Value Correction).**

**Within the <thinking> tags, provide a detailed explanation of your calculation process and reasoning. Then, output the following results within the <result> tags:**

**The ratio of predicted value to actual value for each month.**

**Whether calibration is needed for each month (Yes/No).**

**The calibration coefficient for each month (if applicable).**

**The System Calibration Value (Value Correction).**

**<thinking>**

**[Here, detail your calculation process and reasoning.]**

**</thinking>**

**<result>**

**[Here output the monthly ratios, whether calibration is needed, calibration coefficients (if applicable), and the System Calibration Value.]**

**</result>**

**(5) Vaccine Prompt Template**

**You are a vaccine usage data analyst. Your task is to calculate the final monthly vaccine usage based on given monthly predictions and system correction values for the same vaccine, then compile the results into a complete table and record it in a CSV file.**

**Here is the monthly prediction data:**

**<monthly_predictions>**

**Vaccine Name, Monthly Prediction, Date**

**...**

**</monthly_predictions>**

**Here are the system correction values:**

**<value_correction>**

**Vaccine Name, Value Correction, Date**

**...**

**</value_correction>**

**The calculation formula is: Final Predicted Value = Predicted Value × Correction Value.**

**First, explain the calculation process in detail within the <thinking> tag. Then organize the data in the following table format:**

**Month Predicted Value Correction Value Final Predicted Value**

**[Month 1] [Predicted Value 1] [Correction Value] [Final Value 1]**

**[Month 2] [Predicted Value 2] [Correction Value] [Final Value 2]**

**... ... ... ...**

**Finally, convert the table into CSV format with comma-separated values and headers in the first row.**

**Write the CSV content inside the <csv_content> tag.**

**<thinking>**

**[Explain the calculation process here]**

**</thinking>**

**<csv_content>**

**[Enter CSV file content here]**

**</csv_content>**

2) If this acceptance ratio formally reflects “forecast within ±X% of realized demand,” please specify X and provide a brief rationale (e.g., local procurement tolerances). A parenthetical like “25/35 years-by-vaccine cells met the ±5% criterion (71.43%)” would make the number self-explanatory.

*Response: We thank the reviewer for this valuable comment. We agree that the definition of the acceptance ratio in our original manuscript was not sufficiently clear. As suggested, we have now revised the manuscript to explicitly specify the tolerance threshold (X%) and have adopted the recommended parenthetical format to make the results self-explanatory.*

*The clarification has been incorporated into the manuscript in* lines 286-288*. The revisions now clearly state that the acceptance ratio reflects forecasts within ±X% of realized demand, along with the rationale for choosing this threshold. The results are also presented in the suggested format for enhanced clarity.*

*We believe these revisions have significantly improved the precision and interpretability of our results.*

**lines 286-288:**

**- Acceptance Ratio: forecast falling within ±X% of the realized demand. In this study, the threshold was set at ±5%, as determined by the procurement tolerance range specified by the Shanghai Municipal Center for Disease Control and Prevention (CDC).**

3) Confirm time-respecting evaluation and absence of leakage: Providing a single sentence confirming that training uses only past data and that evaluation is strictly out-of-sample by year (or a rolling-origin scheme) would address typical concerns for time-series studies. If you chose simple year-wise holdouts because procurement is planned annually, noting that operational rationale would be helpful.

*Response: We thank the reviewer for raising this critical point regarding time-respecting evaluation, which is essential for ensuring the validity of our time-series analysis. We sincerely apologize for any lack of clarity in our original manuscript.*

*In response, we have now added a explicit statement in the main text (*lines 178-179*) to confirm that our model training strictly uses only past data and that the evaluation is performed in a strictly out-of-sample manner using a rolling-origin scheme. This approach rigorously prevents any data leakage. Furthermore, as suggested, we have also provided a detailed explanation of the rolling-origin evaluation procedure in the Appendix, which includes the operational rationale behind our design choice.(Appendix C)*

*We believe these revisions have thoroughly addressed the concern and unequivocally demonstrate the robustness of our evaluation methodology.*

**lines 178-179:**

**A rolling-origin scheme was adopted for model training; the specific implementation details are provided in Appendix C.**

**supplement appendix:**

**Appendix C: Methodology of the Multi-step Monthly Rolling-Forecast Evaluation**

**C.1. Rationale and Objective**

**This appendix details the multi-step monthly rolling-forecast scheme employed for the temporal evaluation of the ALLMVP model. This approach simulates a realistic operational scenario where the model is updated with newly forecasted data on a monthly basis to predict subsequent months, thereby rigorously testing its multi-step forecasting capability in a pseudo-productive environment. The final annual aggregates are derived from the summation of these monthly predictions, aligning with the cumulative nature of annual vaccine procurement planning.**

**C.2. Experimental Setup and Data Partitioning**

**The evaluation framework is based on a clear temporal split:**

**• Initial Training Set: Data from January 2014 to December 2017.**

**• Sequential Testing Periods: Data from January 2018 to December 2022. The model generates forecasts for each year within this period through an iterative, expanding-window process.**

**C.3. Step-by-Step Forecasting Procedure**

**The core of the methodology involves a monthly roll-forward mechanism within each forecast year, as conceptually illustrated in Figure C1.**

**Iteration 1: Forecasting for the Year 2018**

**This process begins with the initial training window and proceeds monthly:**

1. **Forecast January 2018: The ALLMVP model is trained on data from Jan 2014-Dec 2017 and generates a prediction for January 2018.**
2. **Expand Training Window (Pseudo-Expansion): The predicted value for January 2018 is incorporated into the training dataset. This creates an expanded training set spanning Jan 2014 - Dec 2017 + Predicted Jan 2018.**
3. **Forecast February 2018: The model is retrained on this expanded set to predict February 2018.**
4. **Iterate Monthly: Steps 2 and 3 are repeated for each subsequent month (March 2018, April 2018, ..., December 2018). At each step, the training window is expanded to include all previous months of 2018, albeit with their predicted values.**
5. **Annual Aggregation: After the December 2018 prediction is made, the 12 monthly predicted values for 2018 are summed to produce the final annual forecast for 2018.**

**Iteration 2: Forecasting for the Year 2019**

**The process continues with a updated baseline:**

**• The training window is now reset to the actual historical data from January 2014 to December 2018 (which includes the actual values for 2018, not the predicted ones from Iteration 1).**

**• The entire monthly procedure described in Iteration 1 is repeated to generate monthly forecasts for 2019, which are then summed to produce the annual forecast for 2019.**

**Subsequent Iterations:**

**This pattern continues—using all available actual data up to the end of the previous year as the baseline for the next year's monthly forecasting cycle—until the entire test period (2018-2022) is covered.**

**C.4. Advantages and Operational Rationale**

**• Simulates Sequential Decision-Making: This method realistically mimics a scenario where planners must make decisions for the next period based on the best available forecast, even before actual data is confirmed.**

**• Rigorous Multi-step Test: It represents a challenging and robust test of the model's ability to maintain accuracy over a long forecasting horizon without being frequently corrected with actual data.**

**• Prevents Data Leakage: The evaluation for any given year uses only data available strictly prior to that year, ensuring a pure out-of-sample test.**

**Appendix Figure 4: Schematic diagram of the multi-step monthly rolling-forecast procedure**

**Appendix Table 5: Summary of the Multi-step Rolling-Forecast Evaluation**

| **Forecast Year** | **Baseline Training Data (Actuals)** | **Forecasting Mechanism** | **Annual Forecast Derivation** |
| --- | --- | --- | --- |
| **2018** | **Jan 2014 - Dec 2017** | **12 sequential monthly forecasts, each using pseudo-expanded training data** | **Sum of 12 monthly predictions** |
| **2019** | **Jan 2014 - Dec 2018** | **12 sequential monthly forecasts, each using pseudo-expanded training data** | **Sum of 12 monthly predictions** |
| **...** | **...** | **...** | **...** |
| **2022** | **Jan 2014 - Dec 2021** | **12 sequential monthly forecasts, each using pseudo-expanded training data** | **Sum of 12 monthly predictions** |

4) Heterogeneity and practical safeguards: Because volatility usually can differ by vaccine, a small table or figure summarizing per-vaccine error distributions (and any systematic over- or under-forecasting) would be useful to program managers deciding where to add buffers.

*Response: We thank the reviewer for this valuable suggestion regarding the importance of analyzing heterogeneity across different vaccines. We agree that summarizing per-vaccine error distributions is crucial for providing practical insights to program managers.*

*In response to this comment, we have now included an analysis of the forecasting error distributions and any systematic biases for each individual vaccine. This analysis can be found in the main text in* lines 464-475*. Additionally, as suggested, we have provided a detailed tabular summary (Appendix Table 4) in the Appendix, which clearly presents the error metrics and systematic over- or under-forecasting tendencies for each vaccine. This addition enhances the practical utility of our findings by directly informing decisions on where to allocate safety buffers.*

*We believe these revisions effectively address the reviewer's concern and significantly strengthen the practical relevance of our study.*

**lines 464-475:**

**Furthermore, the ALLMVP model provides concrete guidance for stockpile management. The error analysis for individual vaccines is presented in Appendix Table 4. The model demonstrated high accuracy for vaccines with stable demand patterns, such as the Hepatitis B vaccine (mean absolute percentage error, MAPE = 2.416%), with no significant systematic bias. In contrast, a clear systematic under-forecasting trend was observed for the Japanese encephalitis vaccine (mean bias error, MBE = -467 doses; MAPE = 5.6%), indicating a more challenging demand pattern. Based on these findings, public health program managers could maintain lower safety stock levels for the Hepatitis B vaccine. For the Japanese encephalitis vaccine, however, we recommend adding a buffer stock of approximately 5–10% to the predicted demand to mitigate the risk of shortages. Such differentiated inventory strategies are crucial for optimizing the overall efficiency and resilience of the vaccine supply chain.**

**Appendix:**

**Appendix Table 4: Summary of forecast error distributions by vaccine type (2018-2022)**

**(a) Year-by-Year Forecast and Error Values**

|  | **Year** | **True Demand (doses)** | **Predicted Demand (doses)** | **Absolute Error** | **Percentage Error (%)** | **Bias** |
| --- | --- | --- | --- | --- | --- | --- |
| **A1** | **2018** | **18907** | **19737** | **830** | **4.39** | **830** |
|  | **2019** | **17135** | **16743** | **392** | **2.29** | **-392** |
|  | **2020** | **17187** | **17316** | **129** | **0.75** | **129** |
|  | **2021** | **17078** | **17359** | **281** | **1.65** | **281** |
|  | **2022** | **16843** | **17349** | **506** | **3.0** | **506** |
| **A3** | **2018** | **22325** | **23055** | **730** | **3.27** | **730** |
|  | **2019** | **17400** | **17220** | **180** | **1.03** | **-180** |
|  | **2020** | **20453** | **20497** | **44** | **0.22** | **44** |
|  | **2021** | **21239** | **22935** | **1696** | **7.99** | **1696** |
|  | **2022** | **9014** | **9127** | **113** | **1.25** | **113** |
| **A4** | **2018** | **12802** | **13496** | **694** | **5.42** | **694** |
|  | **2019** | **10240** | **10284** | **44** | **0.43** | **44** |
|  | **2020** | **11503** | **11996** | **493** | **4.29** | **493** |
|  | **2021** | **5866** | **6386** | **520** | **8.86** | **520** |
|  | **2022** | **4212** | **3844** | **368** | **8.74** | **-368** |
| **A5** | **2018** | **33231** | **35331** | **2100** | **6.32** | **2100** |
|  | **2019** | **31651** | **31740** | **89** | **0.28** | **89** |
|  | **2020** | **32136** | **33284** | **1148** | **3.57** | **1148** |
|  | **2021** | **28925** | **30004** | **1079** | **3.73** | **1079** |
|  | **2022** | **28636** | **30280** | **1644** | **5.74** | **1644** |
| **A6** | **2018** | **30405** | **30869** | **464** | **1.53** | **464** |
|  | **2019** | **27227** | **27988** | **761** | **2.8** | **761** |
|  | **2020** | **27611** | **27215** | **396** | **1.43** | **-396** |
|  | **2021** | **23243** | **23461** | **218** | **0.94** | **218** |
|  | **2022** | **19032** | **20442** | **1410** | **7.41** | **1410** |
| **A7** | **2018** | **20720** | **21216** | **496** | **2.39** | **496** |
|  | **2019** | **18054** | **17971** | **83** | **0.46** | **-83** |
|  | **2020** | **17234** | **14016** | **3218** | **18.67** | **-3218** |
|  | **2021** | **14182** | **14016** | **166** | **1.17** | **-166** |
|  | **2022** | **11929** | **12563** | **634** | **5.31** | **634** |
| **A8** | **2018** | **18765** | **19550** | **785** | **4.18** | **785** |
|  | **2019** | **15479** | **15908** | **429** | **2.77** | **429** |
|  | **2020** | **13494** | **13556** | **62** | **0.46** | **62** |
|  | **2021** | **13659** | **13702** | **43** | **0.31** | **43** |
|  | **2022** | **10816** | **11506** | **690** | **6.38** | **690** |

**(b) Aggregate Forecast Error Metrics (2018-2022)**

| **Vaccine Type** | **Sample Size (years)** | **MAE (doses)** | **MAPE (%)** | **MBE (doses)** | **Analysis of Systematic Bias** |
| --- | --- | --- | --- | --- | --- |
| **A1** | **5** | **427.6** | **2.416** | **270.8** | **Significant over-forecasting- Consistent over-prediction by ~271 doses on average** |
| **A3** | **5** | **552.6** | **2.752** | **480.6** | **Pronounced over-forecasting- Strongest bias among all vaccines** |
| **A4** | **5** | **423.8** | **5.548** | **276.6** | **Clear over-forecasting- Higher MAPE suggests greater relative error** |
| **A5** | **5** | **1212** | **3.928** | **1212** | **Perfect systematic over-forecasting- MBE = MAE indicates all errors are over-predictions** |
| **A6** | **5** | **649.8** | **2.822** | **491.4** | **Substantial over-forecasting- Second strongest positive bias** |
| **A7** | **5** | **919.4** | **5.6** | **-467.4** | **Systematic under-forecasting- Only vaccine showing consistent under-prediction** |
| **A8** | **5** | **401.8** | **2.82** | **401.8** | **Perfect systematic over-forecasting- All predictions are over-estimates** |

**Notes: MAE: Mean Absolute Error; MAPE: Mean Absolute Percentage Error; MBE: Mean Bias Error;**

**Analysis of Systematic Bias**

**The analysis of Mean Bias Error (MBE) reveals distinct systematic forecasting patterns across different vaccine types, which has direct implications for inventory management planning. The results can be categorized into three groups:**

**Pronounced Systematic Over-forecasting (MBE > 0):The majority of vaccines (A1, A3, A4, A5, A6, A8) exhibit significant positive MBE values. This indicates a consistent tendency of the model to over-predictdemand. Notably, for vaccines A5 and A8, the MBE is equal to their MAE (e.g., A5 MBE = 1,212; MAE = 1,212), which implies that for every forecast period, the predicted value was higherthan the true value by approximately 1,212 doses. This strong over-forecasting bias suggests that procurement planners could potentially reduce buffer stocksfor these vaccines to minimize wastage and cost, as the model already builds in a substantial upward buffer.**

**Systematic Under-forecasting (MBE < 0):In contrast, vaccine A7 demonstrates a clear negative MBE (-467.4), signifying a consistent under-predictionof demand. This is a critical finding from a risk management perspective. Under-forecasting poses a higher operational risk than over-forecasting, as it can directly lead to vaccine shortages. For vaccine A7, it is advisable to add a safety bufferto the model's predictions to mitigate the risk of stockouts.**

**Relationship between MAE and MBE:The closeness of the MAE and MBE values for several vaccines (especially A5 and A8) further confirms that the forecast error is not random but is predominantly driven by a consistent directional bias. The lower MAPE values for most vaccines (e.g., 2.416% for A1) indicate that the absolute size of the error is relatively small compared to the demand volume, yet the systematic nature of the bias is a key insight for optimizing inventory levels.**

**In summary, the model exhibits a generally conservative bias, predominantly over-forecasting demand. However, the notable exception of vaccine A7 requires a targeted strategy to prevent shortages. These findings enable program managers to implement differentiated inventory policies rather than a one-size-fits-all approach.**

Additional minor edits:

1)Define acronyms on first use; standardize vaccine names across text/tables.

*Response: We thank the reviewer for this important reminder regarding academic rigor and presentation clarity. We sincerely apologize for these oversights in our original manuscript.*

*In response, we have thoroughly reviewed the entire manuscript to ensure that all acronyms are defined upon their first use. Furthermore, we have standardized the names of all vaccines across the main text, tables, and appendices to eliminate any inconsistency. Additionally, we have included a list of abbreviations (*lines 544-559*) for the readers' convenience.*

*We believe these efforts have significantly improved the readability and professionalism of our manuscript.*

**lines 544-559:**

**List of Abbreviations:**

**A-LR: Logistic Regression with LLM Correction**

**A-RF: Random Forest with LLM Correction**

**A-LSTM: LSTM with LLM Correction**

**AI: Artificial Intelligence**

**ALLMVP: Adaptive Large Language Model for Vaccine Prediction**

**LLM: Large Language Model**

**LLMVP: Large Language Model for Vaccine Prediction**

**LR: Logistic Regression**

**LSTM: Long Short-Term Memory**

**MMR: Measles-Mumps-Rubella**

**NIP: National Immunization Program**

**RF: Random Forest**

**RMSE: Root Mean Square Error**

**SIPIMS: Shanghai Immunization Program Information Management System**

**WHO: World Health Organization**

2) Improve some figure captions (axes, units) and consider adding prediction intervals or error bars where appropriate.

*Response: We are grateful to the reviewer for this highly insightful suggestion, which significantly contributes to enhancing the clarity and professionalism of our manuscript. The reviewer's expertise in data presentation is greatly appreciated.*

*In direct response to this valuable comment, we have thoroughly reviewed and optimized all figure captions throughout the manuscript. The improvements are focused on making the captions more descriptive and reader-friendly, ensuring they effectively guide the interpretation of the figures.*

*We sincerely thank the reviewer for this constructive feedback, which has undoubtedly improved the overall quality of our work.*

To conclude, this is a significant, practice-oriented contribution. The method is simple, the motivation is strong, and the results are encouraging. With the clarifications above, mainly presentation and reproducibility notes, I believe the paper will be ready for publication.

**Reviewer #2:**

This paper presents an interesting and useful application for AI predictive models in regards to vaccine demand prediction, particularly given the importance of effective resource allocation for public health systems from the onset of the COVID-19 Pandemic. The use of multiple models tested on multiple vaccine types across several years, along with multiple evaluation metrics, indicates robust experimentation and methodological detail.

However, there remain areas for improvement in regards to the comparative analysis of the models's predictive performance in Section 3. While a sufficiently strong amount of data and evidence in regards to the acceptance and deviation ratios of various models is referenced in the evaluation and comparison of the tested models, the analysis currently seems relatively subjective. Statistical testing could be used to demonstrate statistical significance in the performance of LLM models against traditional models, as well as adaptive value-correlated models against their non-adaptive counterpart models. Parameter estimation techniques could also be used to better estimate and generalize the predictive strength of the models.

*Response: We thank the reviewer for this insightful and critical comment regarding the comparative analysis of model performance. We agree that incorporating statistical significance tests and parameter estimation techniques would provide a more objective and rigorous foundation for comparing the models, and we acknowledge this as a limitation of our current study.*

*In direct response to this valuable suggestion, we have explicitly discussed this point in the "Limitations and Future Work" section of the manuscript (*lines 513-520*). In these lines, we state that while our evaluation presents strong empirical evidence, future work should include statistical testing to formally establish significance between model classes (e.g., LLM-based vs. traditional models) and employ parameter estimation techniques to better generalize the predictive strength of the approaches.*

*We sincerely appreciate the reviewer's guidance, which has not only improved the self-critical awareness of our present manuscript but also provides a clear and valuable direction for our subsequent research.*

**lines 513-520:**

**Statistical Validation: While the comparative analysis of acceptance and deviation ratios provides substantial evidence of performance differences, the incorporation of formal statistical testing (e.g., to demonstrate the significance of differences between LLM-based and traditional models, or between adaptive and non-adaptive counterparts) and parameter estimation techniques (e.g., to compute confidence intervals for accuracy metrics) would further strengthen the validity and generalizability of the conclusions. Implementing such rigorous statistical analyses represents a valuable direction for future work to provide an even more robust evaluation of the model's predictive strength.**

Furthermore, a more detailed discussion and literature review on the strength of LLM models in Section 1 could be given in order to provide a more developed reasoning behind the stronger performance of the LLM models against the traditional models as validated in Section 3.

*Response: We thank the reviewer for this excellent suggestion, which helps to strengthen the theoretical rationale behind our work. We agree that providing a more developed reasoning in the introduction is crucial for contextualizing the empirical results presented in Section 3.*

*In response, we have enriched the literature review and discussion in the Introduction (*Lines 141-149*) to elaborate on the inherent strengths of LLMs that justify their stronger performance. Specifically, we have added a discussion highlighting:*

*（1）The powerful temporal pattern recognition and extrapolation capabilities of LLMs, attributing this to core architectural components like the self-attention mechanism that effectively capture long-range dependencies in data.*

*（2）The exceptional few-shot learning ability of LLMs, facilitated by their prompt-based framework, which allows them to integrate complex information and adapt to limited-data scenarios effectively.*

*We believe these additions have significantly strengthened the logical flow of our manuscript by establishing a clear a priorirationale for the superior performance of our LLM-based approach, which is subsequently validated by the experimental results.*

**lines 141-149:**

**To address these challenges, our study employs large language models (LLMs) for vaccine prediction, motivated by their inherent advantages in temporal analysis. Unlike conventional models, the core architecture of LLMs, such as self-attention mechanisms, enables more flexible capture of long-range dependencies within the data. Furthermore, their strong few-shot learning capabilities and prompt-based framework allow for effective integration of complex information and adaptation to limited data scenarios. These theoretical strengths position LLMs to achieve significantly superior performance compared to traditional methods**
